# Genome-wide DNA methylation analysis of *Metarhizium anisopliae* during tick mimicked infection condition

**DOI:** 10.1186/s12864-019-6220-1

**Published:** 2019-11-11

**Authors:** Nicolau Sbaraini, Reinaldo Bellini, Augusto Bartz Penteriche, Rafael Lucas Muniz Guedes, Ane Wichine Acosta Garcia, Alexandra Lehmkuhl Gerber, Marilene Henning Vainstein, Ana Tereza Ribeiro de Vasconcelos, Augusto Schrank, Charley Christian Staats

**Affiliations:** 1 Centro de Biotecnologia, UFRGS, Porto Alegre, RS Brazil; 2 Laboratório Nacional de Computação Científica, LNCC, Petrópolis, RJ Brazil; 3 Rede Avançada em Biologia Computacional, RABICÓ, Petrópolis, RJ Brazil

**Keywords:** *Metarhizium*, *Metarhizium anisopliae*, Virulence determinants, DNA methylation, Secondary metabolites, Cell wall morphogenesis

## Abstract

**Background:**

The *Metarhizium* genus harbors important entomopathogenic fungi. These species have been widely explored as biological control agents, and strategies to improve the fungal virulence are under investigation. Thus, the interaction between *Metarhizium* species and susceptible hosts have been explored employing different methods in order to characterize putative virulence determinants. However, the impact of epigenetic modulation on the infection cycle of *Metarhizium* is still an open topic. Among the different epigenetic modifications, DNA methylation of cytosine bases is an important mechanism to control gene expression in several organisms. To better understand if DNA methylation can govern *Metarhizium*-host interactions, the genome-wide DNA methylation profile of *Metarhizium anisopliae* was explored in two conditions: tick mimicked infection and a saprophytic-like control.

**Results:**

Using a genome wide DNA methylation profile based on bisulfite sequencing (BS-Seq), approximately 0.60% of the total cytosines were methylated in saprophytic-like condition, which was lower than the DNA methylation level (0.89%) in tick mimicked infection condition. A total of 670 mRNA genes were found to be putatively methylated, with 390 mRNA genes uniquely methylated in the tick mimicked infection condition. GO terms linked to response to stimuli, cell wall morphogenesis, cytoskeleton morphogenesis and secondary metabolism biosynthesis were over-represented in the tick mimicked infection condition, suggesting that energy metabolism is directed towards the regulation of genes associated with infection. However, recognized virulence determinants known to be expressed at distinct infection steps, such as the destruxin backbone gene and the collagen-like protein gene *Mcl1*, were found methylated, suggesting that a dynamic pattern of methylation could be found during the infectious process. These results were further endorsed employing RT-qPCR from cultures treated or not with the DNA methyltransferase inhibitor 5-Azacytidine.

**Conclusions:**

The set of genes here analyzed focused on secondary metabolites associated genes, known to be involved in several processes, including virulence. The BS-Seq pipeline and RT-qPCR analysis employing 5-Azacytidine led to identification of methylated virulence genes in *M. anisopliae*. The results provided evidences that DNA methylation in *M. anisopliae* comprises another layer of gene expression regulation, suggesting a main role of DNA methylation regulating putative virulence determinants during *M. anisopliae* infection cycle.

## Background

Pest activities are one of the major problems associated with farming. The animal rearing and creation, as well the management of farming lands, disrupts the ecological stability that regulates potential pest species [1]. Insects and other arthropods are particularly problematic pests worldwide. In Brazil, where agriculture is the main source of income, insect-pests cause an average annual loss of 7.7% in crop production (US$ 17.7 billion), resulting in the reduction of approximately 25 million tons of food, fiber, and biofuels [2]. Chemical pesticides are still the usual method for arthropod-pests control causing great concern, in view of the known negative side effects to humans, animals and the environment. Thus, the development of safer and environmentally compatible new pest control tools is pivotal [3].

Entomopathogenic fungi are complex organisms that use a myriad of strategies to achieve a successful infection and can be used to control the major arthropod pests of agriculture, as well as vectors of diseases. Among the most commonly entomopathogenic fungi applied in biological control are the species from *Metarhizium* genus, particularly *Metarhizium anisopliae* [2]. The infection cycle of *M. anisopliae* begins when viable conidia attach to the host cuticle. Under favorable conditions, the conidia germinate and develop the appressorium, a specialized infection structure, in order to transpose the host cuticle barrier. Once into the host hemocoel, hyphae differentiate into blastospores, unicellular infection structures that help in host colonization by fungal dispersion, leading the host to death. After host death, the fungus switches for a saprophytic state, in order to consume the host body and produce new conidia [4].

In recent years, genome sequencing, RNA-seq, and comparative genomic analyses have been used for an exploratory view of the genomes and for the discovery of new virulence determinants in *Metarhizium* spp. [5–7]. However, the still limited knowledge about *Metarhizium*-host interactions is one of the factors that limit in-depth entomopathogenic application for control of economic important arthropods species. DNA methylation of cytosine bases is a heritable epigenetic mark and an important mechanism to control gene expression. DNA methylation is regarded as a key and stable mechanism to repress gene transcription [8]. Striking, different isoforms of DNA methyltransferases (DNMTs) are enrolled in the process. These enzymes catalyze the transfer of methyl groups to cytosine bases, leading to the formation of 5-methylcytosine (5mC) [8]. The presence and genome pattern distribution of 5mCs have been explored in several fungal species, including the *Metarhizium robertsii* [9]. Remarkably, 5mC patterns in fungal genomes fluctuate from low levels (1.8% *Ganoderma sinense* [10]) to almost undetectable levels in *Magnaporthe oryzae* (0.22% [11]) and *M. robertsii* (0.38 to 0.42%). However, these lower levels of DNA methylation still significantly affect the fungal fitness. In *M. oryzae*, DNMT null mutant strains showed defects in asexual reproduction. In addition, such strains displayed an imbalance of transposable elements silencing [11]. Moreover, *M. robertsii* DNMT knockout strains showed similar defects in asexual reproduction (e.g., defects in conidial production), vegetative growth, and virulence [12].

In view of DNA methylation importance in several organisms, including species in the *Metarhizium* genus, it is reasonable to expect that this epigenetic mark can regulate major steps, as well as virulence determinants, during entomopathogenic infection. Thus, we explored DNA methylation patterns in *M. anisopliae* during two very distinct conditions: fungal growth over cattle-tick cuticles (i. e., mimicking an infection condition that have been useful for induction of virulence determinants) and in complete rich medium (i. e., a saprophytic growth condition with abundance of nutrients). Additionally, we compared the Bisulfite sequencing (BS-Seq) results with previous RNA-seq data obtained in the same experimental conditions and the results were further confirmed employing quantitative reverse transcription PCR (RT-qPCR) in the presence or absence of DNMT inhibitor. The results here demonstrate that more regions are methylated under the mimicked infection condition. Additionally, we suggest a putative role for DNA methylation repressing putative virulence factors during the transition between virulent and saprophytic states during *M. anisopliae* infection cycle.

## Results

### Global mapping of DNA methylation in rich medium (saprophytic-like condition) and tick cuticles (mimicked infection condition)

In order to understand the impact of DNA methylation in *M. anisopliae* distinct lifecycles, a BS-seq was conducted using a mimicked infection condition (*M. anisopliae* growth in Tick Cuticles; 48hTC) and a control, saprophytic-like condition (*M. anisopliae* growth in Rich Medium; 48hRM). The experiments herein analyzed followed the recommendations of the Standards and Guidelines for Whole Genome Shotgun Bisulfite Sequencing of the NIH Roadmap Epigenomics Mapping Consortium, which suggests the use of at least two biological replicates with an average coverage of at least 30 times [13]. Two biological replicates were used from each condition and, after trimming and performing quality controls, an average of 6.5 million and 3.36 million clean paired-end reads were obtained for 48hRM and 48hTC, respectively. Mapped sequencing coverage had an average of 51 times for 48hRM and 31 times for 48hTC. The cytosines present in genome were detected with a high coverage (91.03% for 48hRM and 85.17% for 48hTC). Notably, a higher proportion of the identified methylated sites was found in the 48hTC condition (0.89% of total cytosines detected) compared to the 48hRM condition (0.60% of total cytosines detected). For the 48hRM condition, most methylated sites were found at CHH residues (60.44%), followed by CpG sites (21.25%) and CHG sites (18.31%). For the 48hTC condition, a similar scenario was found, with 61.88% of methylated sites occurring at CHH residues, followed by CpG (20.23%) and CHG (17.89%) sites (Table 1).

**Table 1:** Patterns of putative 5mCs sites distribution in the conditions evaluated

	CpG	CHG	CHH	TOTAL
48hTC	0.50%* (20.23%**)	0.55%* (17.89%**)	1.15%* (61.88%**)	0.89%***
48hRM	0.40%* (21.25%**)	0.45%* (18.31%**)	1.85 %* (60.44%**)	0.60%***

*Percentage of putative 5mCs sites across the genome normalized by the total number of Cs in a context-dependent fashion;

**Percentage of residues predominance among the putative 5mCs sites identified;

*** Percentage of putative 5mCs sites across the genome normalized by the total number of Cs in genome

### Identification and functional prediction of putatively methylated mRNA genes

**Fig. 1:**
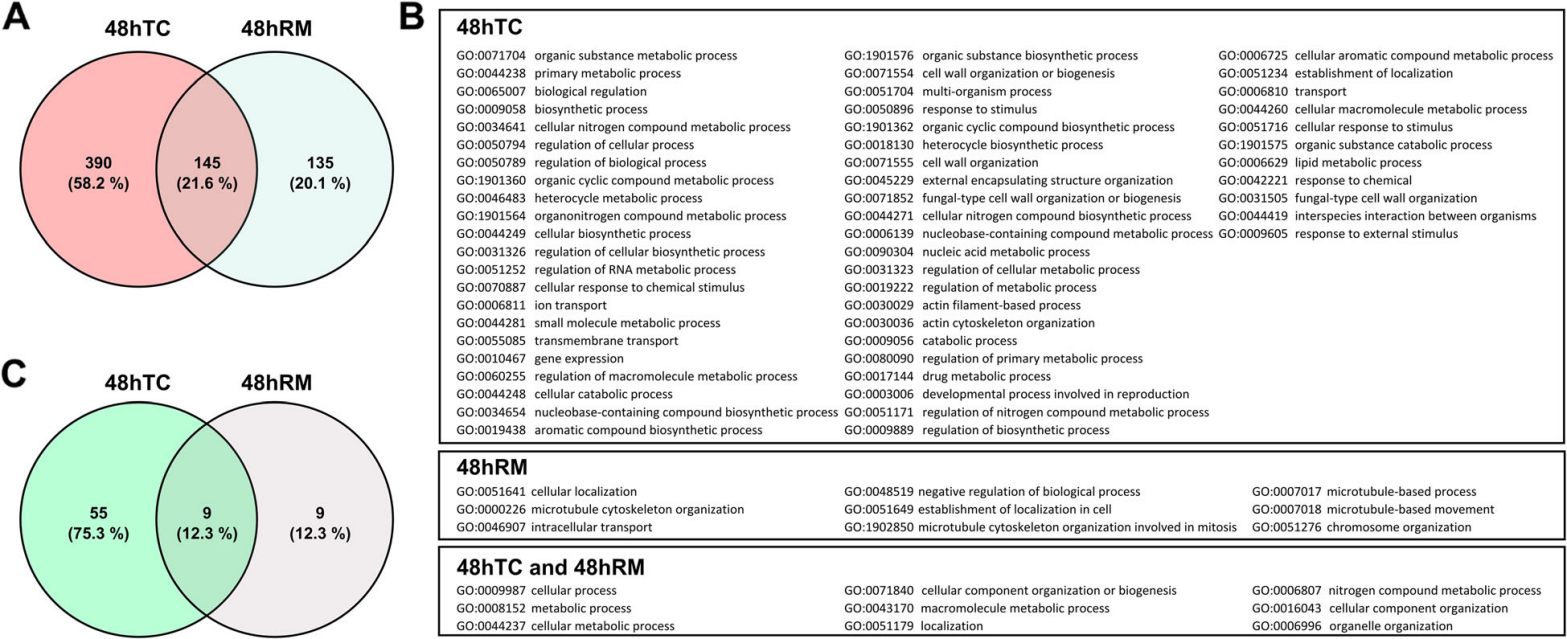
Putatively methylated mRNA genes and GO enrichment analysis. **a** Venn diagram depicting the set of methylated genes in 48hTC and 48hRM. **b** Seventy-three GO terms were over-represented, with 55 GO terms in the 48hTC condition, 9 GO terms in the 48hRM condition and 9 GO terms in both conditions. **c** Venn diagram depicting the set of enriched GO terms in 48hTC and 48hRM

A stringent criterion was used to evaluate potentially methylated genes. It consisted in the identification of 5mCs in the open reading frames (ORFs) of each gene and their respective 500 bp flanking regions. Only sequences spanning an average of 20 5mCs identified were considered methylated. In both conditions (48hTC and 48hRM), a total of 670 protein-coding genes attended such criteria (Fig. 1a and Additional file 1). Accordingly, besides more methylated sites, the 48hTC condition showed more putative methylated genes (i. e., 390 mRNA genes were uniquely methylated in the 48hTC condition) when compared with 48hRM (i. e., 135 mRNA genes were uniquely methylated in the 48hRM) with 145 mRNA genes methylated in both conditions (Fig. 1a). However, no differences could be found in the content of methylation in these 145 putatively methylated genes when the two conditions were compared.

To functionally characterize the set of mRNA genes putatively methylated, the predicted proteins were analyzed for the presence of conserved domains using the NCBI Conserved Domain Database (CDD). A small fraction (~ 5.1%) of the putatively methylated protein coding genes did not presented an associated predicted domain (Additional file 2). Furthermore, the three most abundant domains refer to Adenylate forming domain (cl17068), ABC ATPase superfamily (cl 25,403), and Acyl transferase domain (cl08282), all of which related to synthesis of secondary metabolites (Additional File 2). Gene Ontology (GO) enrichment analysis revealed that 73 GO terms were over-represented among the methylated mRNA genes (Fig. 1b and Additional file 3). Notably, 55 GO terms were uniquely found in the 48hTC condition, 9 GO terms were uniquely found in the 48hRM condition and 9 GO terms were found in both conditions (Fig. 1b and c). There were several GO terms over-represented in 48hTC linked to cell remodeling (GO:0071554, GO:0071555, GO:0071852, and GO:0031505), and regulation of response to stimulus (GO:0065007, GO:0050794, GO:0050789, GO:0031326, GO:0070887, GO:0060255, GO:0050896, GO:0031323, GO:0019222, GO:0003006, GO:0051234, GO:0051716, GO:0042221, GO:0044419, and GO: 0009605 (Fig. 1b). Furthermore, GO terms linked to cytoskeleton morphogenesis appeared on both conditions: actin process and organization in 48hTC (GO:0030029 and GO:0030036) and microtubule organization in 48hRM (GO:1902850, GO:0000226, GO:0007017 and GO:0007018), although the GO terms are not shared between both condition (Fig. 1b). Thus, the results indicate that DNA methylation can regulate genes related to fungal cell morphogenesis and stimuli processing in *M. anisopliae* and DNA methylation can, potentially, affect the transition between specialized infection structures during arthropod colonization.

### DNA methylation and secondary metabolite backbone genes

Secondary metabolites (SMs) are small molecules with a myriad of biological activities and applications. In fungi, the genes required for biosynthesis of SMs are usually found arranged in co-regulated biosynthetic gene clusters (BGCs), which contains backbone genes (e. g., polyketide synthases [PKS], non-ribosomal peptide synthetases [NRPS], hybrids [PKS-NRPS] and terpene cyclases [TCs]), as well as adjacent genes that assist in metabolite maturation [14, 15]. Notably, GO terms associated to SM biosynthesis were found in 48hTC (GO:0019438 [aromatic compound biosynthetic process], GO:0044281 [small molecule metabolic process], GO:0017144 [drug metabolic process], GO:1901362 [organic cyclic compound biosynthetic process] and GO:1901360 [organic cyclic compound metabolic process]).

**Fig. 2:**
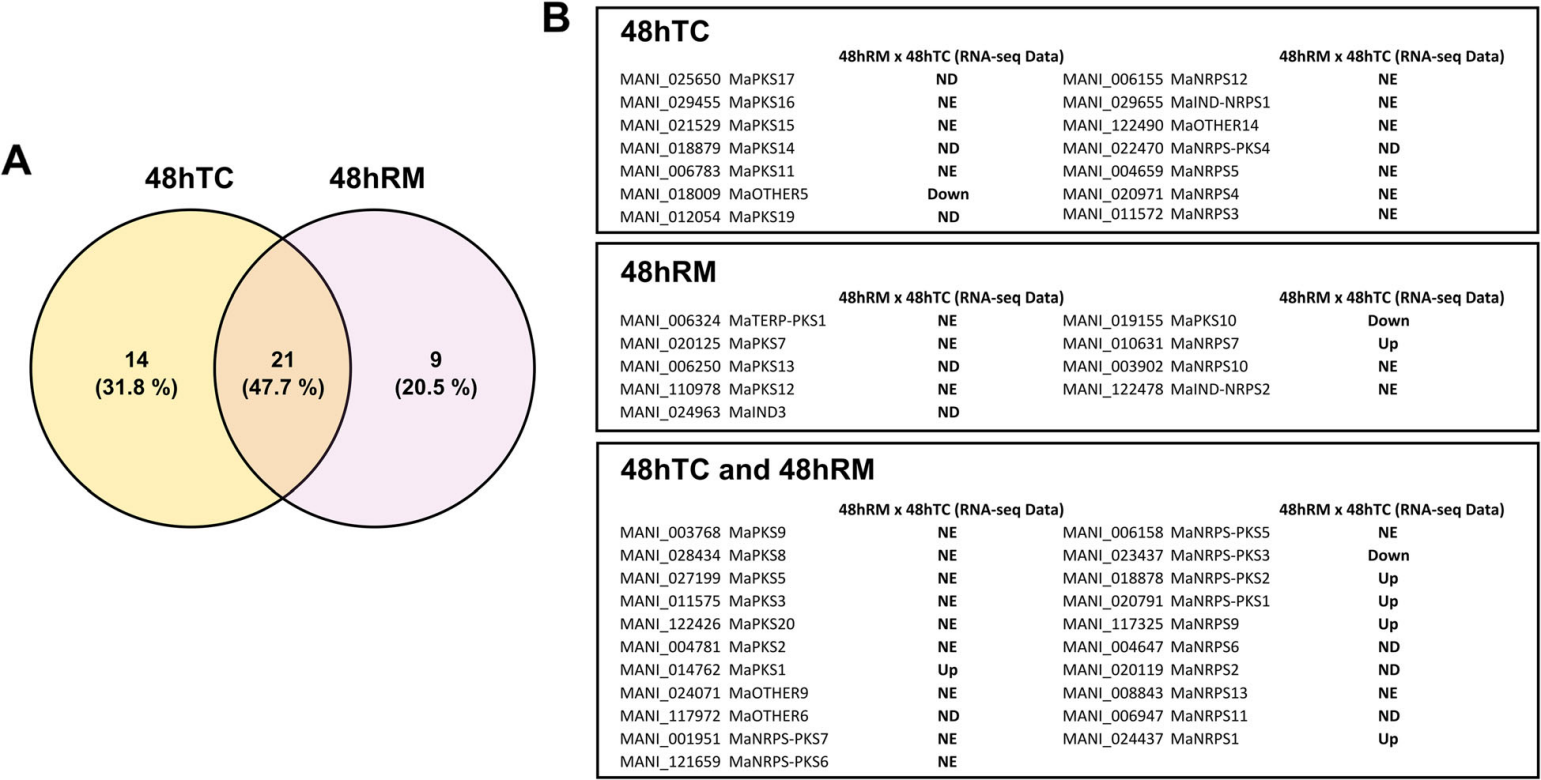
The impact of DNA methylation on secondary metabolite backbone genes. **a** Venn diagram depicting the set of putatively methylated SM backbone genes in 48hTC and 48hRM. **b** Expression and differential expression profile of the 44 putatively methylated backbone genes on the comparison 48hRM x 48hTC performed by [6]. BGC/backbone gene nomenclatures were extracted from [7]. Up: up-regulated; Down: down-regulated; ND: no difference; NE: not expressed

Thus, the methylation pattern of the backbone genes from 73 BGCs found in *M. anisopliae* strain E6 was inferred from the BS-Seq results (Fig. 2; BGC/Backbone gene nomenclature follows our previous report [7]). Additionally, since backbone gene decreased expression led to decreased compound synthesis [16], the exploration of the methylated pattern of the backbone gene, as well as transcription activity of the backbone gene can be a indicator of BGC active/inactive state. In the 48hTC condition, 14 backbone genes were putative methylated, while 9 backbone genes were putative methylated in 48hRM condition and 21 backbone gene were putative methylated in both conditions, which correspond to near 60% of the total of BGCs found in *M. anisopliae* genome (Fig. 2a). As we previously generated RNA-seq data using the same experimental design used to acquire the BS-seq data (i. e., 48hRM and 48hTC, each in biological duplicates) [6], the expression of the putative methylated backbone genes was inferred from the RNA-seq data, looking for possible correlations between methylated state and expression profile. Ten out of 44 BGCs ( 22.7%) displayed detectable expression in the RNA-seq data (RPKM ≥2), but there were no statistical differences (ND) between conditions (Fig. 2b and Additional file 4). Three out of 44 ( 6.8%) putative methylated BGCs were down-regulated (Down) in the RNA-seq data (Fig. 2b and Additional file 4). Additionally, from those 73 BGCs originally identified, 15 BGCs were up-regulated (Up) in the comparison 48hRM x 48hTC, indicating a bigger expression in the 48hTC condition, as previously described [7]. Six out of 44 (13.6%) putative methylated BGCs were among those up-regulated in the RNA-seq data (Fig. 2b and Additional file 4). Noteworthy, 25 out of 44 BGCs (56.8%) did not have detectable expression (NE) in the RNA-seq data in both conditions (RPKM < 2) (Fig. 2B and Additional file 4). However, it is important to notice that nearly half of the BGCs (among the 73 identified) were silent under the conditions evaluated in the RNA-seq [7]. In this way, the results suggest that DNA methylation can be important to regulate the silent state of these biosynthetic pathways.

### Pattern of expression of the putative methylated mRNA genes inferred from the RNA-seq data

We extended the evaluation of the patterns of expression using the RNA-seq data for all putative methylated protein coding genes found. To be classified as differentially expressed, the genes must display a fold change of at least of 1 with FDR corrected *p*-value lower than 0.01, when considered the comparison between the conditions herein analyzed (48hRM and 48hTC). A total of 474 out of 670 (~ 70%) putative methylated protein coding genes displayed detectable expression in the RNA-seq data (RPKM ≥2), but there were no statistical differences between conditions (48hRM x 48hTC) (Additional file 4). This contrasts with the subset of putative methylated BCGs backbone genes, which did not display detectable expression in the RNA-seq data (Fig. 2b). Noticeably, the methylated genes that fall in the ND category are abundant in all conditions (~ 73% for protein coding genes only methylated in the 48hTC condition, ~ 66% for protein coding genes only methylated in the 48hRM condition and ~ 67% for protein coding genes methylated in both conditions) (Additional file 4). Additionally, a total of 79 out of 670 (~ 12%) putative methylated protein coding genes did not have detectable expression in the RNA-seq data (RPKM < 2), a total 40 out of 670 (~ 6%) putative methylated mRNA genes were down-regulated in the RNA-seq data and 77 out of 670 (~ 11%) putative methylated mRNA genes were up-regulated in the RNA-seq data (Additional file 4). Although, the vast majority of putative methylated mRNA genes were expressed, a clear pattern of down-regulation or up-regulation linked to DNA methylation could not be observed. As previously reported by Li and coworkers (2017) for *Metarhizium robertsii*, DNA methylation at the putative promoter or gene ORFs does not always imply transcriptional changes. Additionally, promoter methylation can even enhance gene expression [9].

### Evaluation of putative methylated genes expression using RT-qPCR

To validate the results from BS-seq, we selected seven genes to further analyze by RT-qPCR. These genes belong to three different categories: (I) genes putatively methylated; (II) genes with methylation sites but under the established cut-off of 20; and (III) DNMT genes from *M. anisopliae* genome (whose orthologs were previously functionally characterized in *M. robertsii* [12]). The putatively methylated mRNA genes have been chosen based on the RNA-seq data and putative importance on *Metarhizium* biology. MANI_024437 is the backbone gene for the destruxin BGC (MaNRPS1) [7], which was strongly up-regulated in the comparison 48hRM x 48hTC (Fig. 2b and Additional file 4) and it was putatively methylated in 48hRM and 48hTC conditions (Additional file 1). MANI_023437 is another backbone gene, which codes for a protein putatively enrolled in the biosynthesis of a xenolozoyenone-like metabolite [MaNRPS-PKS3]) [7]. MANI_023437 was down-regulated in the comparison 48hRM x 48hTC (Fig. 2b) and it was putative methylated in 48hRM and 48hTC conditions(Additional file 1). MANI_111160 codes for a collagen-like protein (*Mcl1*), a known virulence determinant [17] and MANI_026638 codes for a putative chitin synthase enrolled in cell wall morphogenesis. Both genes (MANI_111160 and MANI_026638) did have detectable expression in the RNA-seq data (RPKM ≥2), but there was no statistical difference between the experimental conditions (Additional file 4). Additionally, while MANI_026638 was putative methylated in 48hRM and 48hTC, MANI_111160 was only putative methylated in the 48hTC condition (Additional file 1). As a control for the methylation cut-off, MANI_017257, which codes a putative exo-beta-1,3-glucanase from family 17 of glycoside hydrolases, was included in the analysis. To gain information on how the DNA methylation can affect gene expression when the fungus was grown on tick cuticles as the sole carbon source (the condition with the greatest number of putative methylated mRNA genes), DNMT activity was inhibited by adding 5-Azacytidine (5-AZA) to the cultures.

**Fig. 3:**
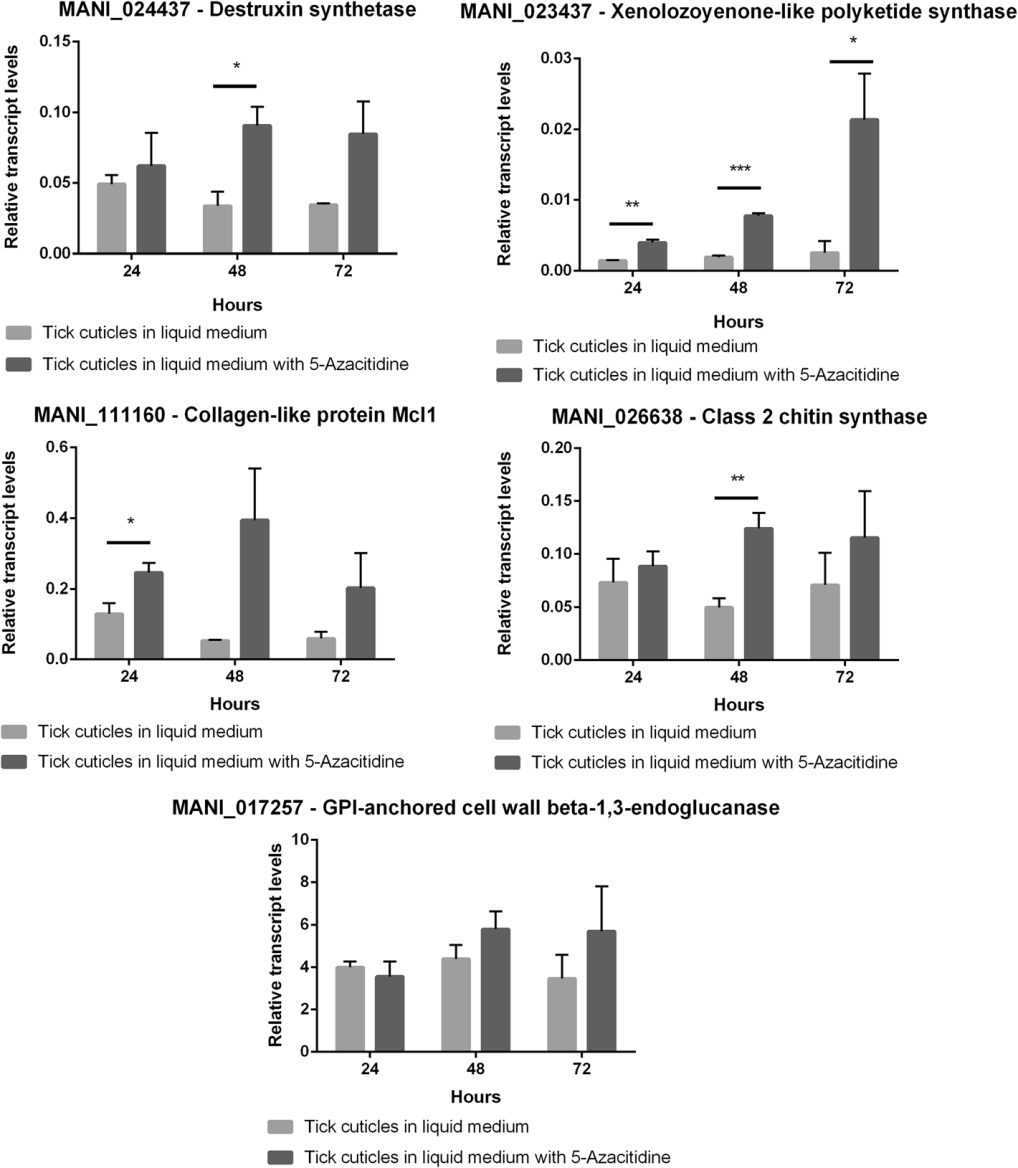
The impact of 5-Azacytidine treatment on methylated genes expression. Quantitative real time RT-PCRs of MANI_024437 (Destruxin synthetase); MANI_023437 (Xenolozoyenone-like polyketide synthase); MANI_111160 (Collagen-like protein *Mcl1*); MANI_026638 (Class 2 chitin synthase) and MANI_017257 (GPI-anchored cell wall beta-1,3-endoglucanase) were performed after growth of *M. anisopliae* E6 with *R. microplus* cuticles, as the sole carbon and nitrogen source, for 24, 48 and 72 h with and without 200 mM of 5-azacytidine (an DNMT inhibitor) supplementation. The results were processed according to 2^-ΔCt^ method and relative transcript levels were normalized with beta-tubulin (MANI_018534). Data are shown as the mean ± SD from three experimental replicates of three biological replicates. * *p* < 0.05; ** *p* < 0.01

**Fig. 4:**
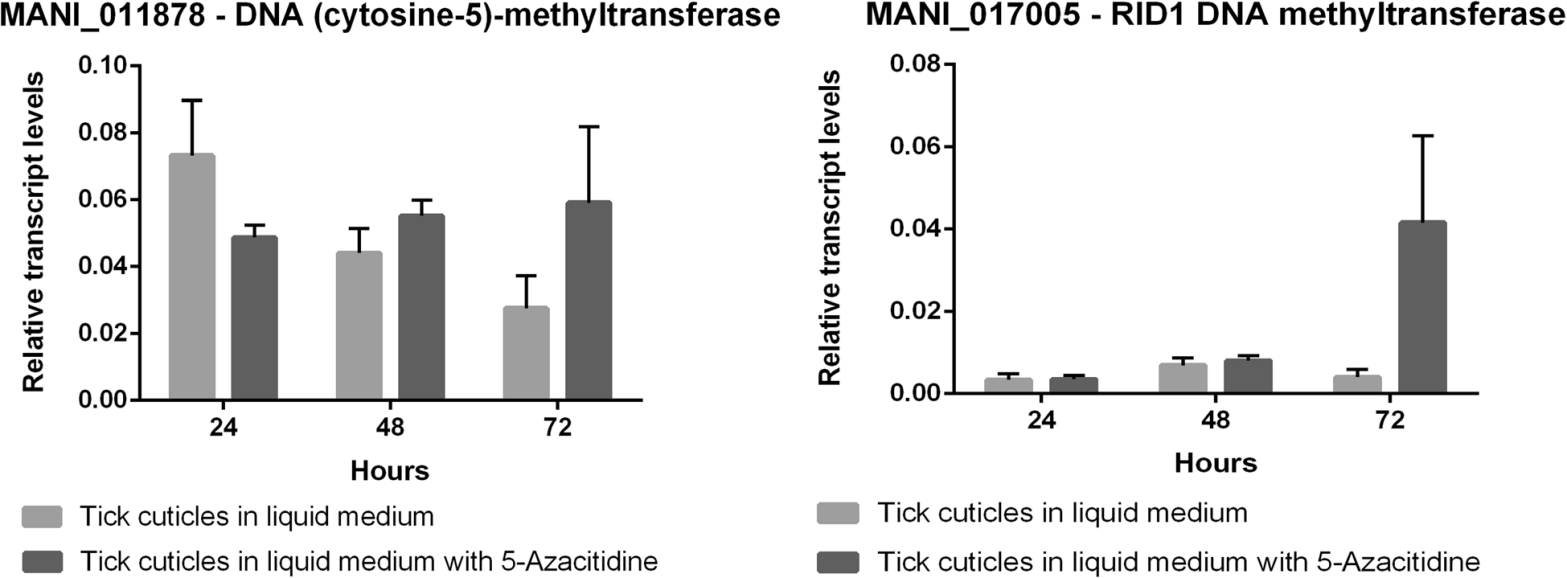
The impact of 5-Azacytidine treatment on DNMTs expression. Quantitative real time RT-PCRs of MANI_011878 (DNA cytosine-5-methyltransferase) and MANI_017005 (RID1 DNA methyltransferase) were performed after growth of *M. anisopliae* E6 with *R. microplus* cuticles, as the sole carbon and nitrogen source, for 24, 48 and 72 h with and without 200 mM of 5-azacytidine (an DNMT inhibitor) supplementation. The results were processed according to 2^-ΔCt^ method and relative transcript levels were normalized with beta-tubulin (MANI_018534). Data are shown as the mean ± SD from three experimental replicates of three biological replicates

The gene expression patterns of the seven chosen genes were explored using three incubation periods (24, 48, and 72 h), which spans the period between the early interaction between fungal cells and tick cuticles to the establishment of the infection. The results obtained with 5-AZA treatment support the BS-Seq results (Fig. 3). For all chosen identified methylated genes, 5-AZA treatment led to increased expression in, at least, one of the incubation times analyzed (Fig. 3). For MANI_111160, 5-AZA treatment led to increased expression in 24 h (Fig. 3). For MANI_024437 and MANI_111160, 5-AZA treatment led to increased expression in 48 h (Fig. 3). Strikingly, for MANI_023437, 5-AZA treatment led to increased expression in all times explored (Fig. 3). Remarkably, no statistically significant differences were found for MANI_017257 with and without 5-AZA treatment (Fig. 3) supporting the cut-off previously established. Moreover, both DNMTs analyzed did not show statistically significant expression differences with the 5-AZA’s treatment, showing that, at least, when the fungus is grown with tick cuticles as the sole carbon source, a potential negative feedback, induced by DNA methylation, may not happen (Fig. 4).

## Discussion

Entomopathogenic fungi and arthropod-pathogenic fungi from *Metarhizium* genus are cosmopolitan species that can survive on soil (as a saprophyte) as well as infect arthropods (as a pathogen) and plants (as an endophyte) [4, 18]. The adaptation to different niches and hosts needs different repertoires of genes, effector molecules and cellular structures. During a saprophytic growth under a carbon and nitrogen rich medium (e. g., MCc), the expression of virulence determinants, only required for host infection, should be, theoretically, not induced. On the other hand, during pathogen-arthropod interaction, to attain a successful infection, *Metarhizium* spp. should switch between different specialized infection structures, up-regulate virulence determinants as well as keep a tight control of endogenous resources to avoid death by starvation.

The impact of the epigenetic machinery, specifically DNA methylation, in the lifecycle of fungal species from *Metarhizium* genus have started to be addressed in *M. robertsii* [9]. Exploring the changes in the methylation pattern between the conidia and mycelia stages, Li and coworkers (2017) showed that approximately 0.38% of the total number of cytosines were putatively methylated in conidia, while 0.42% of the total number of cytosines were putatively methylated in mycelia [9]. However, the impact of DNA methylation in the infection process was not evaluated in *M. robertsii*. In the experiments conducted here, we started to address this problem, employing a mimicked infection condition that has been used before and a saprophytic-like condition as a control [6, 19–21]. Noteworthy, previous results have shown that virulence determinants were up-regulated in the mimicked infection condition [6, 7]. Remarkably, when compared to the results of Li and coworkers (2017), more putatively methylated sites were found in *M. anisopliae* strain E6 (0.60–0.89%), suggesting that different conditions can greatly influence the methylation patterns. Furthermore, species-specific factors can also influence methylation, as previously observed, the methylation pattern between different species can differ markedly [22]. Noteworthy, although more putative methylated sites were found in *M. anisopliae*, the proportion of putative methylated sites in the CHH, CpG and CHG residues were similar between the *Metarhizium* spp., with ~ 57, 23 and 20% of the methylation sites in CHH, CpG and CHG residues, respectively, in *M. robertsii*; compared to ~ 61, 21 and 18% in *M. anisopliae*.

Furthermore, the BS-Seq results support the impact of DNA methylation modification on the modulation of *M. anisopliae* virulence. It is assumed that, in the presence of glucose, other catabolic pathways and virulence determinants should be repressed in *M. anisopliae*, as there is no need to express these genes in a nutrient rich condition. Whereas, in the infection condition, these pathways would be available, in view of host’s/nutrient’s complexity. However, what we found was the contrary of that hypothesis, with more genes putatively methylated in the infection condition. Among the methylated genes, two well-known virulence determinants were found: the destruxin backbone gene and the collagen-like protein MCL1. MCL1 cotes blastospores and is enrolled in evasion from host immune responses [17]. It is important to note that MCL1 expression is tight controlled during the infection cycle of *M. anisopliae,* with the higher expression occurring during hemolymph colonization [23]. Destruxins are important metabolites produced by fungi from *Metarhizium* genus [24]. These compounds have shown insecticidal activity against several arthropod hosts [24–26] and are essential molecules for infection of some *Metarhizium* species [7, 27]. Moreover, the production of destruxin, evaluated by the expression of the destruxin BGC (MaNRPS1), have previously displayed a temporal pattern of regulation during the mimicked infection condition [7]. In this way, it is feasible to propose that the patterns of 5mCs in virulence genes can be modulated during the infection cycle. However, more experiments are needed to confirm such suggestion.

The influence of chromatin structure and epigenetic machinery on SM biosynthesis is well-known [28, 29]. As several backbone genes enrolled in the biosynthesis of SM are putatively methylated, it is possible that *M. anisopliae* employs DNA methylation as an additional mechanism to control SM-associated gene expression. In line with this assumption, the treatment of fungal cultures with drugs that affects the epigenetic machinery have led to isolation of new SMs in several fungal species [29]. Moreover, the results herein shown that employing 5-AZA led to the up-regulation of at least two SM backbone genes. Collectively, the results suggest that culture treatment with 5-AZA may drive the isolation and characterization of new SMs from *M. anisopliae*.

As the infection cycle progresses, *M. anisopliae* switches through different specialized infection structures (e.g., appressorium and blastospore) and lifestyle states (e. g., entomopathogenic and saprophytic) [4]. These changes imply in transcriptional reprogramming to best-fit the different fungal requirements. Chitin synthesis and degradation are essential steps during fungal cell morphogenesis. Among the methylated genes, a chitin synthase gene was chosen for RT-qPCR evaluation. As the results showed, 5-AZA treatment led to increased expression. Moreover, the importance of the DNA methylation on cell morphogenesis is further supported by the GO analysis, reiteratively demonstrating the importance of this epigenetic modification in the lifecycle and infection of *M. anisopliae*.

## Conclusions

Although several layers of gene regulation exist in *Metarhizium* spp., mimicked infection models (e. g., tick cuticles or hind wings) have been valuable conditions to induce virulence determinants expression and specialized infection structures formation [7, 30–32]. In view of complexity of factors that can influence a successful outcome for *Metarhizium* spp. in the infection process, the expression of virulence determinants can be crucial even when the virulence determinants are potentially energetically expensive, as SMs [33]. In the absence of host’s stimuli, the energetic expenditure should be reallocated. In this scenario, the DNA methylation may be an important mechanism. Furthermore, the DNA methylation can also be important to ensure that a given gene will not be expressed outside a specific phase of infection or specific cellular structure. The results here presented provide an overview of methylation dynamics during a mimicked *Metarhizium*-host interaction, contributing with the already established foundation for this epigenetic mark in *Metarhizium* species.

## Methods

### Fungal culture and maintenance, samples treatment and DNA extraction

*M. anisopliae* strain E6 was originally isolated from *Deois flavopicta* collected in Espírito Santo State, Brazil [34]. This strain was maintained at 28 °C in solid Cove’s Complete Medium (MCc), which contains (w/v) 1% glucose, 0.6% NaNO_3_, 0.15% casein hydrolysate, 0.05% yeast extract, 0.2% peptone and 1.5% Agar. After sterilization, 2% (v/v) of filter-sterilized Salts Solution (2.6% KCl, 2.6% MgSO_4_•7H_2_O and 7.6% KH_2_PO_4_ (w/v)) and 0.04% (v/v) of filter-sterilized Trace Elements Solution (0.04% Na_2_Ba_4_O_7_•7H_2_O, 0.4% CuSO_4_•5H_2_O, 0.01% FeSO_4_, 0.8% Na_2_MbO_4_•7H_2_O, 0.8% MnSO_4_•7H_2_O and 0.8% ZnSO_4_•7H_2_O (w/v)) were added to the sterilized medium prior to use (modified from [35]).

*Rhipicephalus microplus* cuticles were prepared by dissection of ingurgitated females, extensively washed with sterile water and sterilized by autoclaving [31]. Spore suspension (5 × 10^6^ spores per mL^− 1^) was used to inoculate the cuticles by immersion for 30 s. The inoculated cuticles were disposed over 1% water agar plates and maintained for 48 h at 28 °C (mimicked infection condition, Tick Cuticle: 48hTC). Each of the two biological replicates consisted of mycelium grown over approximately 5 g (wet weight) of tick cuticles, which are disposed onto 5 Petri dishes. The comparative control condition was conducted with two biological replicates of *M. anisopliae* grown in 100 ml liquid MCc for 48 h at 28 °C (Control condition, Rich Medium: 48hRM). The resulting mycelia obtained on such conditions were grounded to powder in liquid nitrogen and DNA extraction was carried out through a standard protocol [36]. Extracted DNA was subjected to RNAse treatment and stored at − 20 °C.

### Bisulfite conversion, library construction and sequencing

Before bisulfite conversion, one ug of each DNA sample, referring to the two biological replicates from the two conditions herein analyzed (48hTC and 48hRM) were cleaned up with Agencourt AMPure beads XP (Beckman Coulter). Samples were quantified using a Qubit 2.0 fluorometer (Invitrogen) to estimate the DNA yield. *Escherichia coli* strain ER2925 Non-methylated Genomic DNA (Zymo Research) was spiked in each sample (1 ng of control per 200 ng of sample) to determine the bisulfite conversion efficiency and as a quality control for bisulfite treatment. Bisulfite conversion was carried out with EZ DNA Methylation-Gold™ Kit (Zymo Research) following manufacturer’s instructions. The only exception was the 64 °C incubation step, which was performed for only 1.5 h instead of 2.5 h, in order to avoid DNA degradation. After bisulfite conversion, the samples were quantified using a NanoDrop 2000 spectrophotometer (ThermoFisher Scientific). The sequencing of the libraries was performed with TrueSeq DNA Methylation kit (Illumina) using as input the four bisulfite-treated single-stranded DNA samples. All the steps of the protocol were carried out following manufacturer’s instructions. Quality control of the distinct libraries was performed using the 2100 Bioanalyzer System with the Agilent High Sensitivity DNA Kit (Agilent). The libraries were individually quantified via qPCR using a KAPA Library Quantification Kits for Illumina platforms (KAPA Biosystems). Using adaptors containing distinct indexes, a total of four libraries were pooled together in equimolar amounts and sequenced in a MiSeq sequencing system (Illumina) using MiSeq Reagent Kits to obtain 250 bp paired-end reads.

### Data treatment

The total output reads were deindexed (separation of reads) based upon the unique bar-codes using the BaseSpace pipeline into distinct fastQ files, corresponding to each of the four conditions herein analyzed (two biological replicates for 48hTC and two biological replicates for 48hRM). The raw paired-end reads were visually inspected with FastQC [37], trimmed and subjected to quality control using Trimmomatic [38] . Reads were processed to remove adapter and other illumina-specific sequences (ILLUMINACLIP:TruSeq2-PE.fa:2:20:10), the first 20 nucleotides were cropped (HEADCROP:20), the reads were limited to a size of 225 nucleotides (CROP:225), reads with Phred quality scores lower than 20 in the beginning and 25 in the end were cropped (LEADING:20 TRAILING:25) and only reads greater than 30 were considered (MINLEN:30). The resulting reads were aligned to the *M. anisopliae* strain E6 [6] and *E. coli* strain ER2925 (K12 derivative) genome sequences using Bismark/ Bowtie2 [39, 40]. Genome coverage from each library was calculated using Coverage/Read Count Calculator (http://apps.bioconnector.virginia.edu/covcalc/). In order to distinguish between 5mCs and background, the observed methylation in *E. coli* ER2925 DNA was used as a background control, to provide a false-positive measurement of bisulfite treatment. The error rates, for each replicate, were determined as 0.005, 0.003, 0.006, and 0.005 for 48hRM R1, 48hRM R2, 48hTC R1 and 48hTC R2, respectively. These values represent the probability *p* (false discovery rate) on the binomial distribution *p* (*n*, *p*). Based in such probabilities, the *p*-values were calculated, and the hypothesis’ tests were performed to determine the methylated cytosines, being the significance threshold set to 0.02 [41]. For each sample the bisulfite conversion rate was > 97.4%. After this procedure, the positions of 5mCs was verified in *M. anisopliae* strain E6 genome annotation [6] in order to identify putative methylated genes. A generic definition of gene was used here, which expanded the ORF and the surrounding 500 bp upstream and downstream. Genes were classified as methylated if they had reads mapping for at least in average, from both biological replicates, of 20 5mCs, as previously demonstrated [22]. A diagram, representing the pipeline, is presented in Additional file 7: Fig. S1. All scripts were present in as Additional file 5.

### Domain annotation, gene ontology and RNA-seq data

Genes identified as methylated were evaluated for the presence of conserved domains in their predicted amino acid sequences, using the Conserved Domain Database (CDD) with default parameters (only hits with *e*-value lower than 0.01 were accepted) [42]. Gene Ontology (GO) terms annotation and enrichment was performed using Blast2GO v 5.0 with default parameters: (I) Blast step: *e*-value cut-off 1 × 10^− 10^; the database was the Fungi section of Swiss-Prot; recover of the 10 best hits; (II) Annotation step: cut-off 55; GO weight 5; *e*-value 1 × 10^− 6^; hit filter 500; (III) Enrichment step: only hits with false discovery rate corrected *p*-value smaller or equal to 0.05 were accepted [43]. Veen diagrams were generated with Venny [44].

RNA-seq data (including RPKM values for 48hRM and 48hTC) as well as differential expression (log2-fold change; 48hRM x 48hTC) were recovered from our previously published data [6]. The same experimental design used for the determination of methylation pattern in saprophytic-like (48hRM) and the mimicked infection (48hTC) conditions was employed to determine the genome wide gene expression pattern [6]. Ribosomal RNA-depleted RNA samples isolated from two independent biological replicates from each condition (48hRM and 48hTC) were submitted to RNA-Seq and the differential expression analysis was conducted using HT-Seq and edgeR [6]. For expression analysis, genes with RPKM values ≥ 2 were considered with detectable expression. Genes were considered differentially expressed if the corresponding log2-fold change ratios were ≥ 1 or ≤ − 1, with a 5% false discovery rate corrected *p*-value smaller or equal to 0.01.

### Culture conditions, RNA extraction, sample treatment and RT-qPCR

In order to evaluate the influence of 5mCs presence on gene expression, another independent experiment was conducted. In this experiment, *M. anisopliae* was grown in two different conditions for RNA isolation, employing three different times (24, 48 and 72 h) and three biological replicates for each time. Fungal conidia (5 × 10^6^ spores per mL^− 1^) were inoculated in 100 ml of liquid medium, which consist of *R. microplus* (1% (w/v)), as the sole carbon and nitrogen source, amended or not with 200 mM 5-azacytidine (an DNMT inhibitor). The resulting fungal math was grounded to powder in liquid nitrogen and RNA extraction was carried out using TRIzol reagent protocol (Invitrogen) and treated with RQ DNAse (Promega). Reverse transcription and cDNA synthesis were performed using ImProm-II Reverse transcriptase (Promega) using oligo-dT. The relative expression of four putative methylated genes (MANI_024437 [Destruxin synthetase]; MANI_026638 [Class 2 chitin synthase]; MANI_111160 [Collagen-like protein *Mcl1*]; MANI_023437 [Xenolozoyenone-like polyketide synthase]); one gene (MANI_017257 [GPI-anchored cell wall beta-1,3-endoglucanase]) in which the number of methylation sites did not reach the cut-off (< 20 methylation sites); and the 2 DNMTs found in the genome of *M. anisopliae* strain E6 (MANI_011878 and MANI_017005) were determined by RT-qPCR (StepOne Real-Time PCR System) with an initial step of 95 °C for 10 min, followed by 50 cycles of 95 °C for 15 s, 55 °C for 15 s, and 60 °C for 60 s. A melting curve analysis was performed at the end of the reaction to confirm the presence of single PCR products. The results were processed according to the 2^-ΔCt^ method [45] and relative transcription levels were normalized with beta-tubulin (MANI_018534) gene transcript levels. The primer sequences used in RT-qPCR analysis are listed in Additional file 6.

### RT-qPCR statistical analysis

Data were expressed as mean ± standard deviation (SD) of replicates. All assays were performed in three experiment conditions, with technical triplicate repetitions. The Student’s *t*-test was employed to test for significance between values. Comparisons of means with *p*-values ≤0.05 were considered statistically different.

## Supplementary information

**Additional file 1.** Putatively methylated genes.

**Additional file 2.** CDD analysis.

**Additional file 3.** GO enrichment analysis.

**Additional file 4.** Expression and differential expression profile of the 670 putatively methylated mRNA genes extracted from previously published RNA-seq data.

**Additional file 5.** BS-seq scripts.

**Additional file 6.** The primer sequences used in RT-qPCR analysis.

**Additional file 7: Figure S1.** Flowchart describing the whole BS-seq pipeline.

## Data Availability

The datasets generated during the current study are available in the NCBI’s repository, BioSample accessions SAMN11941230, SAMN11941231, SAMN11941232, SAMN11941233.

## Abbreviations

48hRM: Control condition, Rich Medium
48hTC: Mimicked infection condition, Tick Cuticle
5-AZA: 5-azacytidine
5mC: 5-methylcytosine
BGC: Biosynthetic Gene Cluster
BS-seq: Bisulfite sequencing
CDD: Conserved Domain Database
DNMT: DNA methyltransferase
Down: Down-regulated
GO: Gene Ontology
MCc: Cove’s Complete Medium
ND: No Difference
NE: Not Expressed
NRPS: Non-ribosomal peptide synthetases
PKS: Polyketide synthase
PKS-NRPS: Hybrids (Polyketide synthase/ Non-ribosomal peptide synthetases)
RPKM: Reads Per Kilobase Million
RT-qPCR: quantitative reverse transcription PCR
SD: Standard Deviation
SM: Secondary Metabolite
TC: Terpene Cyclase
Up: Up-regulated
