# Supplementary material for: Genome-wide DNA methylation analysis of Metarhizium anisopliae during tick mimicked infection condition
Source: BMC Genomics. 2019 Nov 11;20:836. doi: 10.1186/s12864-019-6220-1 (PMC6849299; doi:10.1186/s12864-019-6220-1)
Supplement: Supplementary file 6 — Additional file 6. The primer sequences used in RT-qPCR analysis. [file 12864_2019_6220_MOESM6_ESM.docx]

**Additional File 6:** The primer sequences used in RT-qPCR analysis

| **Primer Name** | **Sequence (5’-3’)** | **Target/Goal** |
| --- | --- | --- |
| MANI_026638_qPCR_F1 | CGACGACGCAACTGTATCTATC | Class 2 chitin synthase RT-qPCR |
| MANI_026638_qPCR_R1 | GAGAACCTGACATGAAGGAGTC | Class 2 chitin synthase RT-qPCR |
| MANI_023437_qPCR_F1 | CGAGGAGAAACAGACGAAGAAG | Xenolozoyenone-like polyketide synthase RT-qPCR |
| MANI_023437_qPCR_R1 | ACCTCTGGAAACCATGACATAC | Xenolozoyenone-like polyketide synthase RT-qPCR |
| MANI_017257_qPCR_F1 | ACGATGTCATCTCGGCTATTC | GPI-anchored cell wall beta-1,3-endoglucanase RT-qPCR |
| MANI_017257_qPCR_R1 | GTACTGGTCGATGGTCTTCTTC | GPI-anchored cell wall beta-1,3-endoglucanase RT-qPCR |
| MANI_024437_qPCR_F1 | GCAGCTCGGTTCAAGTTTATTC | Destruxin synthetase RT-qPCR |
| MANI_024437_qPCR_R1 | GAGATGGACCAACAGCAGAGTAG | Destruxin synthetase RT-qPCR |
| MANI_111160_qPCR_F1 | TCGTTCAGGACAGGAGTAGG | Collagen-like protein Mcl1 RT-qPCR |
| MANI_111160_qPCR_R1 | CTGGTTGACGACGATGATGAG | Collagen-like protein Mcl1 RT-qPCR |
| MANI_017005_MARID1_qPCR_F1 | CGGAGGATGTGGTGATACTTG | RID1 DNA methyltransferase RT-qPCR |
| MANI_017005_MARID1_qPCR_R1 | ATCGGTCAAGTCGATGGTAAAG | RID1 DNA methyltransferase RT-qPCR |
| MANI_011878_MaDIM-2_qPCR_F1 | TCAAAGGGAGGCATTTAGGG | DNA (cytosine-5)-methyltransferase RT-qPCR |
| MANI_011878_MaDIM-2_qPCR_R1 | CCTGTCGTTTGTGAGAGTAGAG | DNA (cytosine-5)-methyltransferase RT-qPCR |
| Tub_F | GTAACCAAATTGGTGCTGCT | Tubulin RT-qPCR |
| Tub_R | CGACGGAGAAAGTGGCCATC | Tubulin RT-qPCR |
