# Supplementary figures and images for: Genome-wide DNA methylation analysis of Metarhizium anisopliae during tick mimicked infection condition
Source: BMC Genomics. 2019 Nov 11;20:836. doi: 10.1186/s12864-019-6220-1 (PMC6849299; doi:10.1186/s12864-019-6220-1)

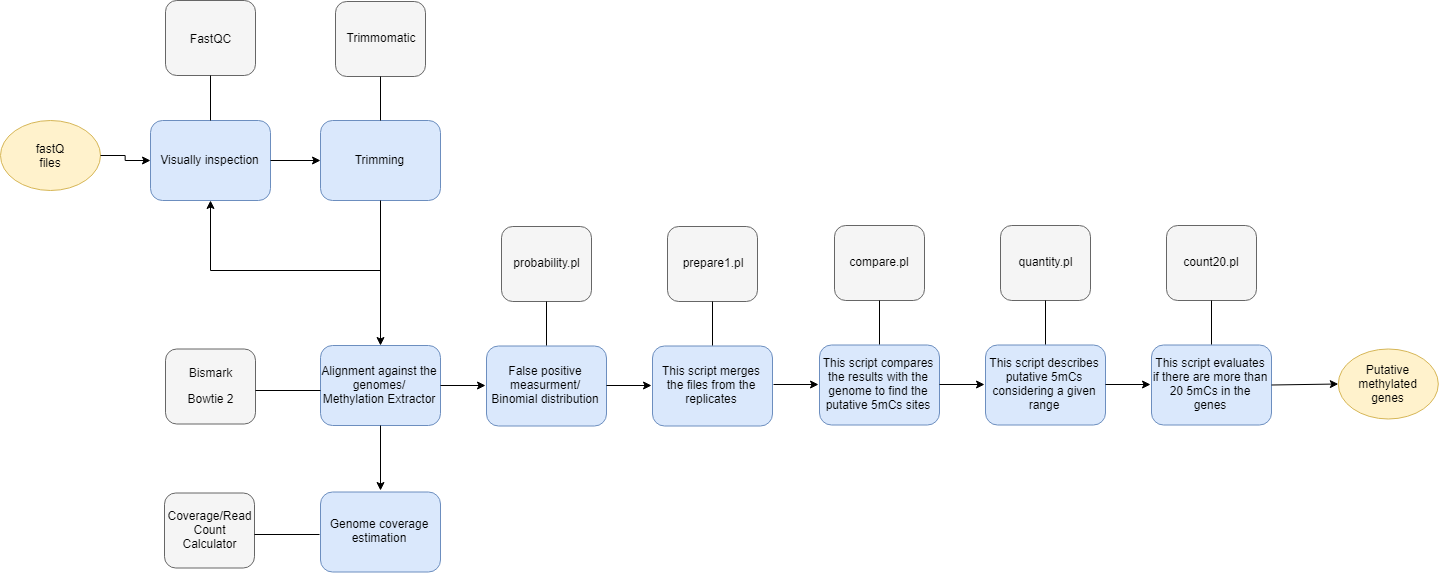

Supplement: Supplementary file 7 — Additional file 7: Figure S1. Flowchart describing the whole BS-seq pipeline. [file 12864_2019_6220_MOESM7_ESM.png]
