# Supplementary material for: Genome-wide DNA methylation analysis of Metarhizium anisopliae during tick mimicked infection condition
Source: BMC Genomics. 2019 Nov 11;20:836. doi: 10.1186/s12864-019-6220-1 (PMC6849299; doi:10.1186/s12864-019-6220-1)
Supplement: Supplementary file 5 — Additional file 5. BS-seq scripts. [file 12864_2019_6220_MOESM5_ESM.zip › Tut_scripts/README.doc]

**Title:** Genome-wide DNA methylation analysis of *Metarhizium anisopliae* during tick mimicked infection condition

**Authors:** Nicolau Sbaraini ^1,2^§, Reinaldo Bellini ^1,3^§, Augusto Bartz Penteriche ^2^, Rafael Lucas Muniz Guedes ^1,3^, Ane Wichine Acosta Garcia ^2^, Alexandra Lehmkuhl Gerber ^3^, Marilene Henning Vainstein ^1,2^, Ana Tereza Ribeiro de Vasconcelos ^1,3^, Augusto Schrank ^1,2^, Charley Christian Staats ^1,2^*

^1^ Rede Avançada em Biologia Computacional (RABICÓ), Petrópolis, RJ, Brazil.

^2^ Centro de Biotecnologia, Programa de Pós-graduação em Biologia Celular e Molecular, Universidade Federal do Rio Grande do Sul, Porto Alegre, RS, Brazil.

^3^ Laboratório Nacional de Computação Científica, LNCC, Petrópolis, RJ, Brazil.

§ Both authors contributed equally to this work.

*Correspondence: [staats@ufrgs.br](mailto:staats@ufrgs.br)

**Additional File 5:** BS-seq pipeline

For more information of reads quality control check [1].

- In order to visualize the reads the FastQC software was employed (fastqc_v0.11.8.zip ) [2], this step allow the visualization of reads for quality control;
- For sequence trimming, Trimmomatic [3] was employed (Trimmomatic-0.36.zip) , in this step, adapters were removed and sequences filtered;

The Trimmomatic parameters and run command used:

java -jar /home/Trimmomatic-0.32/trimmomatic-0.32.jar PE -threads 40 <sequences> ILLUMINACLIP:TruSeq2-PE.fa:2:20:10 HEADCROP:20 CROP:225 LEADING:20 TRAILING:25 MINLEN:30

ILLUMINACLIP: Cut adapter and other illumina-specific sequences from the read.

HEADCROP: Cut the specified number of bases from the start of the read

CROP: Cut the read to a specified length

LEADING: Cut bases off the start of a read, if below a threshold quality

TRAILING: Cut bases off the end of a read, if below a threshold quality

MINLEN: Drop the read if it is below a specified length

- The Bismark software was employed with default parameters (For *M. anisopliae* and *E. coli*, with reads aligned to the respective genomes) [1].

The Bismark parameters and run command used:

bismark_genome_preparation [options] <path_to_genome_folder>

bismark [options] <genome_folder> {-1 <mates1> -2 <mates2>

bismark_methylation_extractor[options] <filenames>

For a better explanation of the parameters, check:

https://www.bioinformatics.babraham.ac.uk/projects/bismark/Bismark_User_Guide.pdf

**In-house scripts:**

- **Binomial distribution (probability.pl)**

In order to distinguish between 5mCs and background, the observed methylation in *E. coli* ER2925 DNA was used as a background control, to provide a false-positive measurement of bisulfite treatment. The error rates, for each replicate, were determined as 0.005, 0.003, 0.006, and 0.005 for 48hRM R1, 48hRM R2, 48hTC R1 and 48hTC R2, respectively. These values represent the probability *p* (false discovery rate) on the binomial distribution *p* (*n*, *p*). Based in such probabilities, the *p*-values were calculated, and the hypothesis’ tests were performed to determine the methylated cytosines, being the significance threshold set to 0.02 [4].

**How to run:**

perl probability.pl

**First interaction:**

Insert your input: (Insert your “.cov” file)

**Output:** archive “answer.csv”. Rename this file to “.cov” for the next analysis.

**Observation**: Repeat this procedure for all samples

- **(prepare1.pl)**

This script converts two “.cov” files (replicates) in a merged “.csv” file. This step is necessary for next script that describe the putatively methylated sequences.

**How to run:**

perl prepare1.pl

**First interaction:**

Insert your first archive: (“.cov file” for the first replicate)

**Second interaction:**

Insert your second archive: (“.cov file” for the second replicate)

**Output:**

A directory with a “.csv “file

- **script compare (compare.pl)**

This script describes the putatively methylation sites in the genome. This needs the **oberdan_gbk.pl** script.

**How to run:**

perl compare.pl

**First interaction:**

Contigs/ Chromosomes total number? (total number of contigs/chromosomes in the genome)

**Second interaction:**

Add the .gbff genome: (Insert your Genbank Genome file containing genome sequence and annotation)

**Output:**

A directory with a “.csv “file

- **script quantity (quantity.pl)**

This script describes the putative 5mCs considering a given range [e.g., -500bp, ORF, +500bp]

**How to run:**

perl quantity.pl

**First interaction:**

Contigs/ Chromosomes total number? (total number of contigs/chromosomes in the genome)

**Second interaction:**

How many base pairs, BEFORE the ORF, do you want? (number of base pairs before the ORF)

**Third interaction:**

How many base pairs, AFTER the ORF, do you want? (number of base pairs after the ORF)

**Output:**

File “FINAL-ANSWER.csv”

**“FINAL-ANSWER.csv”**

Column 1 – Locus Tag

Column 2 – Contig/Chromosome

Column 3 – Number of 5mCs in the first replicate

Column 4 – Number of 5mCs in the second replicate

Column 5 – Initial base pair in the Contig/Chromosome for a given ORF

Column 6 – Final base pair in Contig/Chromosome for a given ORF

- **count20 (count20.pl)**

This script evaluates if there are more than 20 5mCs in the genes

**How to run:**

perl count20.pl

**Output:**

File “methylated.csv”

**“methylated.csv”:**

Column 1 – Locus Tag

Column 2 – Contig/Chromosome

Column 3 – Number of 5mCs in the first replicate

Column 4 – Number of 5mCs in the second replicate

Column 5 – Initial base pair in the Contig/Chromosome for a given ORF

Column 6 – Final base pair in Contig/Chromosome for a given ORF

Column 7 – Average number of 5mCs

**REFERENCES**
